# The Facilitation of Clinical and Therapeutic Discoveries in Myalgic Encephalomyelitis/Chronic Fatigue Syndrome and Related Diseases: Protocol for the You + ME Registry Research Platform

**DOI:** 10.2196/36798

**Published:** 2022-08-10

**Authors:** Allison Ramiller, Kathleen Mudie, Elle Seibert, Sadie Whittaker

**Affiliations:** 1 Solve ME/CFS Initiative Glendale, CA United States

**Keywords:** myalgic encephalomyelitis/chronic fatigue syndrome, long COVID, data acquisition source, postinfectious, longitudinal cohort study, patient powered, COVID-19, chronic fatigue syndrome, longitudinal health data, symptom-tracking app, health application, mobile health, digital health

## Abstract

**Background:**

Myalgic encephalomyelitis/chronic fatigue syndrome (ME/CFS) is a chronic, complex, heterogeneous disease that affects millions and lacks both diagnostics and treatments. Big data, or the collection of vast quantities of data that can be mined for information, have transformed the understanding of many complex illnesses, such as cancer and multiple sclerosis, by dissecting heterogeneity, identifying subtypes, and enabling the development of personalized treatments. It is possible that big data can reveal the same for ME/CFS.

**Objective:**

This study aims to describe the protocol for the You + ME Registry, present preliminary results related to participant enrollment and satisfaction, and discuss the limitations of the registry as well as next steps.

**Methods:**

We developed and launched the You + ME Registry to collect longitudinal health data from people with ME/CFS, people with long COVID (LC), and control volunteers using rigorous protocols designed to harmonize with other groups collecting data from similar groups of people.

**Results:**

As of September 30, 2021, the You + ME Registry had over 4200 geographically diverse participants (3033/4339, 69.9%, people with ME/CFS; 833/4339, 19.2%, post–COVID-19 people; and 473/4339, 10.9%, control volunteers), with an average of 72 new people registered every week. It has qualified as “great” using a net promotor score, indicating registrants are likely to recommend the registry to a friend. Analyses of collected data are currently underway, and preliminary findings are expected in the near future.

**Conclusions:**

The You + ME Registry is an invaluable resource because it integrates with a symptom-tracking app, as well as a biorepository, to provide a robust and rich data set that is available to qualified researchers. Accordingly, it facilitates collaboration that may ultimately uncover causes and help accelerate the development of therapies.

**Trial Registration:**

ClinicalTrials.gov NCT04806620; https://clinicaltrials.gov/ct2/show/NCT04806620

**International Registered Report Identifier (IRRID):**

DERR1-10.2196/36798

## Introduction

### Background to ME/CFS

Myalgic encephalomyelitis/chronic fatigue syndrome (ME/CFS) is a chronic, complex, systemic disease that affects anywhere from 1.5 to 3.4 million people in the United States [1,2], with an estimated annual economic cost of US $36-$51 billion [2]. The etiology of ME/CFS is unknown, but it is associated with infectious triggers or other precipitating events, such as injury, trauma, or exposure to environmental hazards [3]. ME/CFS has not been correlated with age, race, or socioeconomic group; however, 3-4 times as many women as men present with symptoms [3].

The true impact of this disease remains uncertain largely because ME/CFS is commonly misdiagnosed, with primary reasons referenced as a lack of confidence from clinicians and the absence of an established biomarker [4]. Clinicians must rely on a detailed evaluation of the presence of characteristic symptoms, health history, and a physical examination. Although many clinicians are aware of ME/CFS, they often lack essential experience necessary to diagnose this complex disease.

ME/CFS is characterized by debilitating fatigue and other multisystem physical and neurocognitive symptoms, which are exacerbated following minimal physical or mental exertion. Postexertional malaise (PEM) is the hallmark symptom of ME/CFS and is associated with elevated symptom burden and psychological distress [3]. Otherwise, substantial clinical heterogeneity exists among patients, with a range of symptoms that includes orthostatic intolerance (OI), postural tachycardia syndrome (POTS), brain fog, headaches, unrefreshing sleep and other sleep dysfunction, joint pain, and muscle pain/fibromyalgia [3].

Although understanding of biological abnormalities in ME/CFS has increased considerably in recent decades [5-10], the lack of a diagnostic biomarker, complex clinical presentation, and patient heterogeneity have severely hampered progress in the treatment of ME/CFS, causing many patients to have poor outcomes [3,11]. Although there are no treatments approved for ME/CFS, a number of pharmacological and nonpharmacological interventions are used to manage symptoms. There have been a few promising clinical trials undertaken; however, they have not resulted in approved therapies for the ME/CFS population. For example, researchers analyzing data from a phase III trial of rintatolimod found that only a subset of ME/CFS patients defined by relatively short duration of disease (symptom onset within 2-8 years) had improved exercise tolerance in response to rintatolimod [12]. Although this is still an experimental treatment, there is an acute interest in identifying subsets of patients who might be responders [12,13].

Methodological problems, small sample sizes, and selection bias toward those less severely affected by ME/CFS [14] also create persistent roadblocks to progress in the understanding of the disease. An observational study that centralizes clinical data and well-designed patient-reported outcomes collected over time from a large, diverse cohort has the potential to increase research validity and generate new insights.

### The You + ME Patient Registry

Patient registries are invaluable resources for collecting real-world clinical data at a large scale that can then be used for research. They comprise a set of systematically collected and stored data that can be harmonized or shared with other research groups, while ensuring a level of data quality and reliability. Patient registries have been effectively used for other complex, heterogenous diseases, such as the Fox Insight study for Parkinson disease [15]; the IBD Partners Registry [16], where IBD refers to inflammatory bowel disease; and ACCELERATE, an international registry for patients with Castleman disease [17].

In May 2020, the Solve ME/CFS Initiative (Solve M.E.), a nonprofit organization whose mission is to make ME/CFS and other postinfection diseases widely understood, diagnosable, and treatable, launched the You + ME Registry and Biobank (Registry) [18]. The Registry is a secure online data repository where people with ME/CFS, people with related diseases, and control volunteers can enter information on their health.

Given the strong evidence for the viral etiology of ME/CFS and experience from the SARS-CoV-1 pandemic of 2003 [19-21], there is a potential for the current SARS-CoV-2 pandemic to lead to a substantial increase in the number of ME/CFS cases. There is evidence that some people experience long-term effects from COVID-19 (termed “long COVID” [LC]) with a constellation of symptoms reported that are strikingly similar to those reported for ME/CFS [22-27]. The COVID-19 pandemic and emergence of long-term symptoms in some individuals present a unique scientific opportunity to understand factors of resistance and susceptibility to long-term, postviral impacts. In December 2020, in response to the increasing number of individuals with LC, the Registry was adapted and opened up to those who are suffering from the long-term effects of COVID-19 and control individuals who had COVID-19 but do not have long-term effects.

The Registry is designed to be a foundational resource for research, and it is unique for several reasons:

- It integrates a symptom-tracking app that can provide more data points for dynamic/cyclic chronic diseases.
- It is a rigorous, systematic infrastructure for collecting data that were cocreated with the ME/CFS and LC communities.
- The data and patient cohorts are available to all qualified researchers, supporting numerous scientific studies and engaging a network of expertise.
- The Registry is centered around principles of data harmonization and collaboration with other research studies to accelerate the search for causes and therapies.

In this paper, we describe the protocol for the Registry, present preliminary results related to participant enrollment and satisfaction, and discuss the limitations of the Registry as well as next steps.

## Methods

### Human-Centered Design

The creation of the Registry integrated community input and human-centered design (HCD) methodologies [28]. Originally developed in the field of computer science and artificial intelligence, it has been adapted so that engagement with and understanding of the needs of users are common to all design disciplines and can be applied to a range of complex questions, from process optimization to product design [29-31]. We gathered qualitative narratives and quantitative data from multiple stakeholders to inform the Registry and symptom-tracking app product development, user experience, and data collection.

One-on-one unstructured phone interviews were conducted with stakeholders and experts. Interviewees included 4 people with ME/CFS across the disease severity spectrum, 1 care partner, and 3 individuals with expertise in disease registries, informatics, and HCD. Notes from the calls were transcribed and summarized into key themes and insights (Table 1).

**Table 1:** Key insights from HCD^a^ interviews with the ME/CFS^b^ community.

Category of expertise	Key insights
People with ME/CFS	Elevate individuals with ME/CFS to partner/contributor status. Create mechanisms that will enable participation and insights for people with severe ME/CFS. Include measures that will corroborate or add on to the symptom data, including a measure to assess functional status.
ME/CFS clinicians and researchers	Ensure collection of information on autonomic function. Track regularly whether the person’s medications have changed and whether they have been diagnosed with new conditions/diseases.
Informatics	Foster a social component because people who engage socially are more likely to continue to enter data. Provide the ability for participants to report quantified self-data and self-experiment (eg, supplements and medications being used).
HCD	Consider more innovative approaches to enable participation of extremes in your patient population.
Informal caregiver	Create a formal, defined user group involved throughout the cycle of the process.
Community relations expert	Partner with advocacy groups for the community, both national and local chapters. Create an advisory board including active patient advocates to review proposals for research.

^a^HCD: human-centered design.

^b^ME/CFS: myalgic encephalomyelitis/chronic fatigue syndrome.

An online survey collected anonymous perspectives from 251 people with ME/CFS regarding symptoms they experience, the method/frequency of data capture that would work best, and how data should be reported back through a mobile app. The results were compared to a community survey developed by collaborators at Columbia University (unpublished), for a combined response set from over 1200 people with ME/CFS.

Symptoms endorsed by most of the survey respondents (ie, fatigue, PEM, cognitive impairment, unrefreshing sleep) were included in a core set of symptoms that autopopulate for app users. However, community feedback made it clear that capturing a complete range of symptoms would be critical for relevancy of the app to the individual, so we included the ability to add custom symptoms. Although most said they would use the app daily or weekly, about one-third indicated this was too frequent.

To accommodate variation in disease severity and function, we set up the app to allow data capture as often as daily and included communications every 3 days to encourage data entry. We also added screens to track activities (eg, work, social, leisure) in the first build of the mobile app because respondents were highly interested in understanding the links between activities and symptoms.

Over 30 individuals tested a beta version of the registration process and tracking app on their Apple and Android phones—including people with ME/CFS, care partners, researchers, and clinicians. Most beta testers used a template form to provide feedback with yes/no options, numerical ranking scales, and open comment fields. Version 2.0 of the app was developed in response to tester input with enhanced user guidance and prompts, the ability to add narratives through a journal screen, and the addition of a calendar functionality. When the Registry was adapted and opened for the post–COVID-19 cohort, we utilized a similar community testing and feedback process. The user experience and data collection are continuously improved based on feedback from patients and researchers.

### Technical Infrastructure

The Registry data are securely stored in an encrypted database hosted in a cloud-based instance [32]. The database stores data records as documents, which are collated into collections (analogous to tables in a relational database) and linked according to unique identifiers. Participant data are encrypted in transit using industry-standard Transport Layer Security/Secure Socket Layer to protect sensitive information when it is transmitted to and from the front-end apps and backend database. Amazon Cognito is used for user authentication and access control. Strict security and access standards are in place to protect participant data.

The Registry is designed to be a globally shared repository that removes data silos and increases collaboration. The digital tools and services that comprise the registry are setup for Health Insurance Portability and Accountability Act (HIPAA) and General Data Protection Regulation (GDPR) compliance to ensure the highest international standards of data privacy are met and to allow enrollment and collection of self-report survey data from participants worldwide. We plan to work with local partners to ensure regional data privacy and regulatory requirements are fulfilled for the collection of future data types (eg, health care data, biological samples).

### Data Harmonization

The validated data collection instruments within the Registry (see the Data Collection section) are aligned with those used by other researchers and clinicians studying patients with ME/CFS. They include the National Institutes of Health (NIH) National Institute of Neurological Disorders and Stroke (NINDS) Common Data Elements [33] to facilitate aggregation of data across studies. After consulting with community members, additional data fields were included to build a richer understanding of each participant’s health history. The Registry also integrates the NINDS centralized Globally Unique Identifier (often referred to as GUID) solution, a secure tool that generates unique IDs without exposing personally identifiable information (PII) to allow data sharing and collaboration across research groups.

### Participant Recruitment

The Registry is open to all individuals with ME/CFS, those with LC, and other populations, including individuals with other chronic diseases and individuals considered healthy controls. The aim is to enroll a diverse global cohort of participants who are representative of the broader ME/CFS and LC communities and to create the largest-possible global data set to interrogate.

The Registry is promoted via Solve M.E.’s and You + ME’s dedicated @youmeregistry social media channels (Facebook, Twitter, Instagram), on a dedicated online informational website [18], printed newsletters, and email to the Solve M.E. listserv. It is also promoted in webinars and conference presentations.

Additional recruitment is conducted through partners in both ME/CFS and LC by leveraging their social media channels and email listservs. This is particularly important for recruitment to our ME/CFS and LC cohorts, given that many in these communities are connected via robust social media groups [34]. Our LC recruitment strategy specifically is multipronged (Table 2). Outreach is focused on areas with historically high incidences of COVID-19 cases (eg, New York City, New Orleans, and Los Angeles). Future plans for recruitment include referrals from clinicians and health systems.

**Table 2:** Overview of current and planned recruitment strategies for the Registry^a^.

Type of outreach	Target organizations/partners and strategy
Solve M.E.^b^ communication channels	Promote the Registry to our established network via (1) a database of over 34,000 active contacts; (2) organizational and Registry social media accounts with a combined following of 6772 on Twitter, 34,114 on Facebook, and 2256 on Instagram; and (3) our educational webinar series for researchers, clinicians, and patients.
COVID-19 survivor postacute sequelae of COVID-19 (PASC) patient groups	Partner with established groups serving COVID-19 survivors and individuals with PASC, including online forums and support groups on social media, to promote the Registry to their networks.
LC^c^ alliance	Partner with members to create a referral pipeline to the Registry from over 50 science, postviral disease, and patient advocacy and research organizations working together to find answers for LC and postviral illness.
Internet and social media advertising	Google Ads and social media posts directed toward individuals who have experience in COVID-19 and primary care providers who may be treating those with persistent symptoms of COVID-19.
Clinics/health systems	Partner with health systems, clinics, and hospitals serving our populations of interest to provide a postcard that will be handed out to their patients with COVID-19. The postcard will ask about the development of persistent postviral symptoms and direct patients to the Registry for voluntary sign-up.
Membership organizations/trade associations	Partner with health care workers and emergency medical services (EMS) unions; other unionized or nonunionized essential workers, such as large grocery/drug store chains, transit workers, and delivery services; university-based, countrywide student organizations, athletic associations. and student health networks; and medical specialty associations to share the recruitment notices to their membership.

^a^Registry: You + ME Registry and Biobank.

^b^Solve M.E.: Solve ME/CFS Initiative, where ME/CFS refers to myalgic encephalomyelitis/chronic fatigue syndrome.

^c^LC: long COVID.

### Criteria for Selection

The Registry is open to individuals of all genders, with an anticipated gender split between males and females reflective of the gender prevalence of ME/CFS and LC. Adults (aged 18 years and above) are eligible for participation. All races and ethnic origins are included. Although we do not limit enrollment from control volunteers, we will be making every effort to ensure controls are adequately matched to patients by age, sex, race, and other key demographic indicators.

#### People With ME/CFS

People with ME/CFS self-diagnosed or diagnosed by a clinician are eligible to enroll. Many patients with ME/CFS struggle for years before being diagnosed; it has been estimated that up to 90% of people with ME/CFS have not received an official diagnosis from a clinician [35,36]. Rather than exclude participants based on a lack of clinical diagnosis at enrollment, we include these individuals and record the method of diagnosis for each participant. We also have a recurring question about clinician diagnosis to track any changes.

#### People With COVID-19

People who had COVID-19, whether confirmed or not confirmed by a lab test, are eligible to enroll. Access to and reliability of COVID-19 lab tests caused difficulties to confirm an infection, particularly in the early period of the pandemic. Therefore, we allow self-reporting of COVID-19 infection and record the method of initial COVID-19 diagnosis for each participant.

#### Control Volunteers

Control volunteers are made up of individuals without ME/CFS or LC, including individuals considered healthy controls and those with other chronic illnesses (eg, fibromyalgia).

### Symptom Assessment and Algorithm for ME/CFS Case Criteria

For all participants, the Registry scores responses to the UK ME/CFS Biobank (UKMEB) Symptoms Assessment Questionnaire to determine fulfillment of distinct ME/CFS case criteria [37], including the CDC-1994 (Fukuda) criteria [38], the Canadian Consensus criteria [39], the International Consensus criteria [40], the Institute of Medicine criteria [41], and the Oxford criteria [42]. This is to align with what has been proposed for use in clinical practice and currently being used by ME/CFS researchers worldwide [43]. Questionnaire responses are fed to a digitized version of the algorithm licensed from the UKMEB to the Registry, which is coded into the online platform in a separate endpoint that securely runs the data for scoring.

### Data Collection

Participants sign up for the Registry via a secure website interface. Participants are asked to complete an electronic informed consent form for collection of data and to be recontacted for optional biosample collection or other study opportunities. After consenting to the Registry, they are guided through a series of baseline surveys (Table 3), including medical history, diagnosed conditions, symptoms and quality of life, and medication history.

**Table 3:** Surveys used in the Registry^a^ and what they measure.

Survey	Measures
ME/CFS^b^ Disease History^c,d^	Disease-specific history (triggers, onset, disease course)
COVID-19 History^e^	Infection status, acute illness, clinical course
UKMEB^f^ Symptoms Assessment [37]^e,g^	Symptom experience and fulfillment of ME/CFS case definitions
Short Form-36 [44]^e,g^	Health-related quality of life
Karnofsky Performance Status (modified) [45]^e^	Functional status
Multidimensional Fatigue Inventory [46,47]	General fatigue, physical fatigue, reduced motivation, reduced activity, and mental fatigue
Demographics^g^	Basic demographic information, including age, race, ethnicity, income, education, employment status
My Conditions^h^	Diagnosed conditions
My Treatments^h^	Medications, supplements, and other treatments
Family Health History^d^	Information about disorders from which a direct blood relative may or may not have suffered
Beighton Score^d^	A screening tool for hypermobility
Fibromyalgia Impact Questionnaire Revised^d,i^	Physical functioning, work status, depression, anxiety, morning tiredness, pain stiffness, fatigue, and well-being
COVID-19 Vaccination Status	Vaccination status, symptoms (pre-existing and following the vaccine), and reasoning for not getting vaccinated (if indicated)

^a^Registry: You + ME Registry and Biobank.

^b^ME/CFS: myalgic encephalomyelitis/chronic fatigue syndrome.

^c^The survey is only presented to those who indicate they have ME/CFS.

^d^A one-time survey.

^e^The exact same questionnaire asked at follow-up timepoints.

^f^UKMEB: UK ME/CFS Biobank.

^g^Abbreviated/modified version of the questionnaire asked at follow-up timepoints.

^h^A form that can be revised/added to on an ongoing basis.

^i^The survey is only presented to those who indicate they have fibromyalgia.

**Figure 1:**
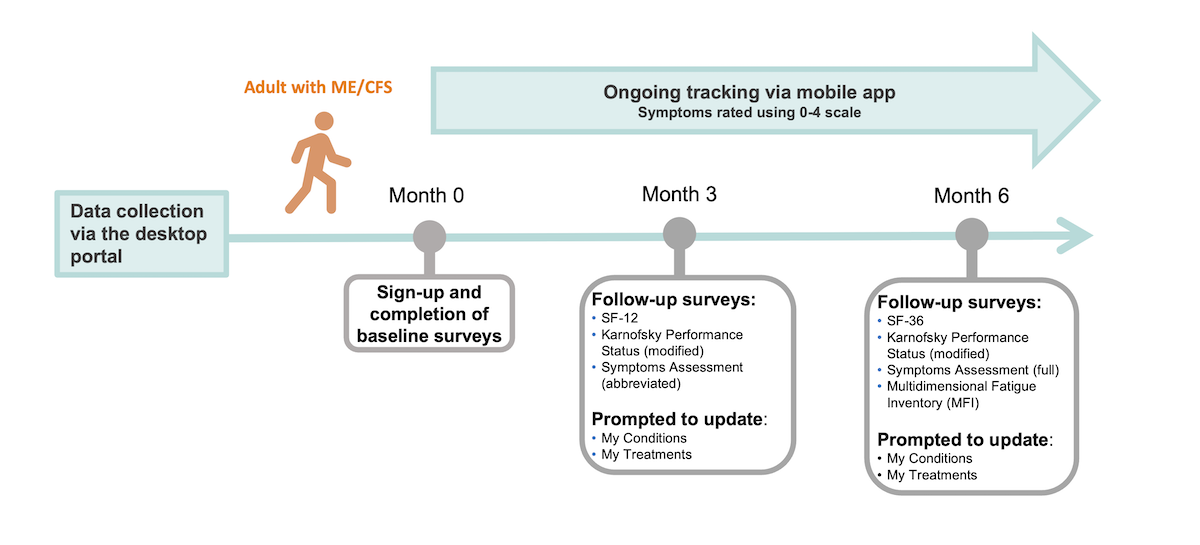
Overview of the first 6 months of longitudinal Registry data collection, which includes electronic surveys administered at enrollment (baseline) and follow-up time intervals in adults with ME/CFS. Individuals with ME/CFS can opt to track their symptoms using a numerical scale from 0 (symptom absent) to 4 (very severe) in a mobile app. Severity scores are defined as follows, according to the DePaul Symptom Questionnaire: 0=symptom not present, 1=mild, 2=moderate, 3=severe, 4=very severe. ME/CFS: myalgic encephalomyelitis/chronic fatigue syndrome; Registry: You + ME Registry and Biobank.

**Figure 2:**
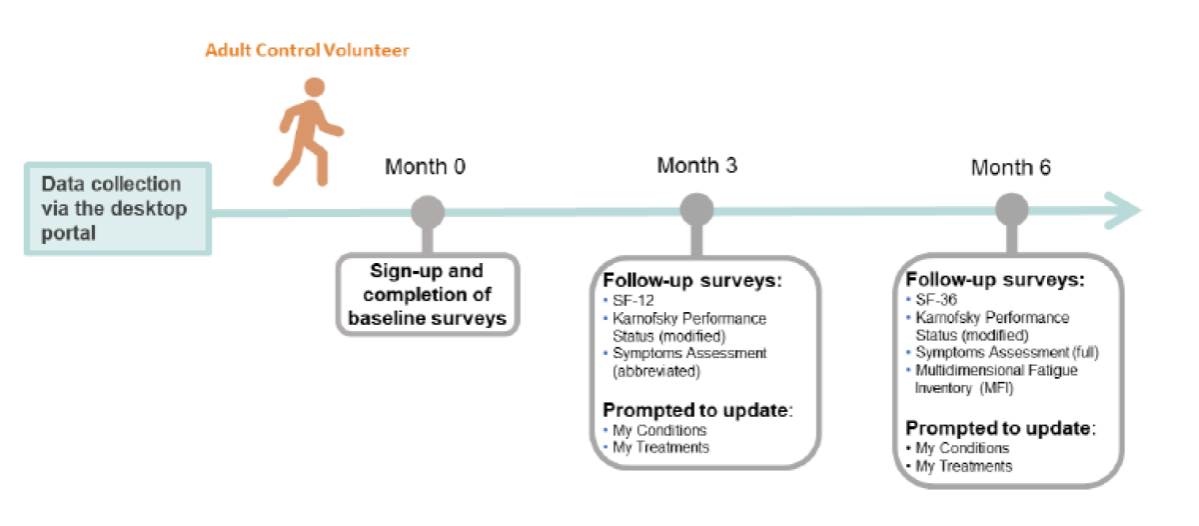
Overview of the first 6 months of longitudinal Registry data collection, which includes electronic surveys administered at enrollment (baseline) and follow-up time intervals in adult control volunteers. ME: myalgic encephalomyelitis; Registry: You + ME Registry and Biobank. SF-12: 12-item Short Form Survey; SF-36: 36-item Short Form Survey.

**Figure 3:**
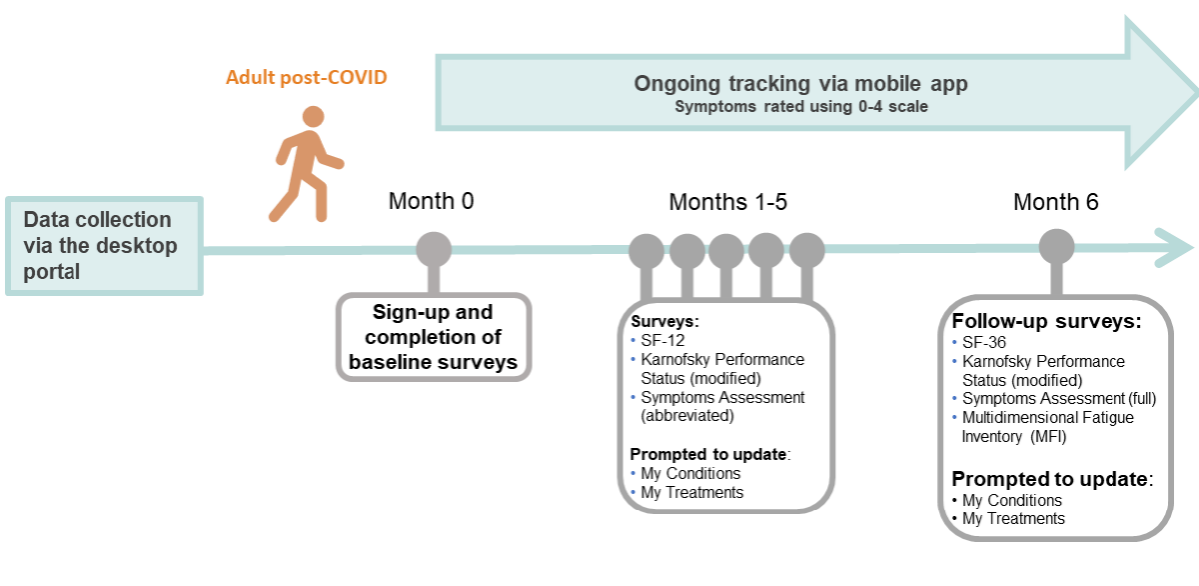
Overview of the first 6 months of longitudinal Registry data collection, which includes electronic surveys administered at enrollment (baseline) and follow-up time intervals in adults post–COVID-19. Individuals can opt to track their symptoms using a numerical scale from 0 (symptom absent) to 4 (very severe) in a mobile app. Severity scores are defined as follows, according to the DePaul Symptom Questionnaire: 0=symptom not present, 1=mild, 2=moderate, 3=severe, 4=very severe. ME: myalgic encephalomyelitis; Registry: You + ME Registry and Biobank. SF-12: 12-item Short Form Survey; SF-36: 36-item Short Form Survey.

Longitudinal characterization is imperative to understanding chronic illnesses that are evolving and have a cyclical nature. Volunteers with ME/CFS and control volunteers are sent email reminders to complete an abbreviated set of surveys every 90 days following registration (Figures 1 and 2). We hypothesize that biologically or clinically distinct factors drive divergent outcomes in post–COVID-19 patients toward complete recovery or long-term sequelae. So, the post–COVID-19 cohort is sent follow-up surveys every 30 days in the initial 6 months and then every 90 days for longer-term follow-up (Figure 3). Through more frequent data collection during this critical period following recent infection, we aim to identify factors driving or predictive of this bifurcation in outcomes.

One-time questionnaires are also deployed through the Registry, in addition to the regular longitudinal assessments. These questionnaires collect cross-sectional data from unique instruments that are not part of the routine longitudinal assessments, and currently include surveys on family health history, joint hypermobility (self-report Beighton), and COVID-19 vaccination experience.

Data are aggregated into a single table and are available as a comma-separated value file. Values of variables are encoded according to a data dictionary, which accompanies each data file and describes the metadata available for each survey question in the routine longitudinal assessment and one-time surveys. The complete data dictionary includes over 800 collected variables and is available upon request.

### Symptom Tracking

**Figure 4:**
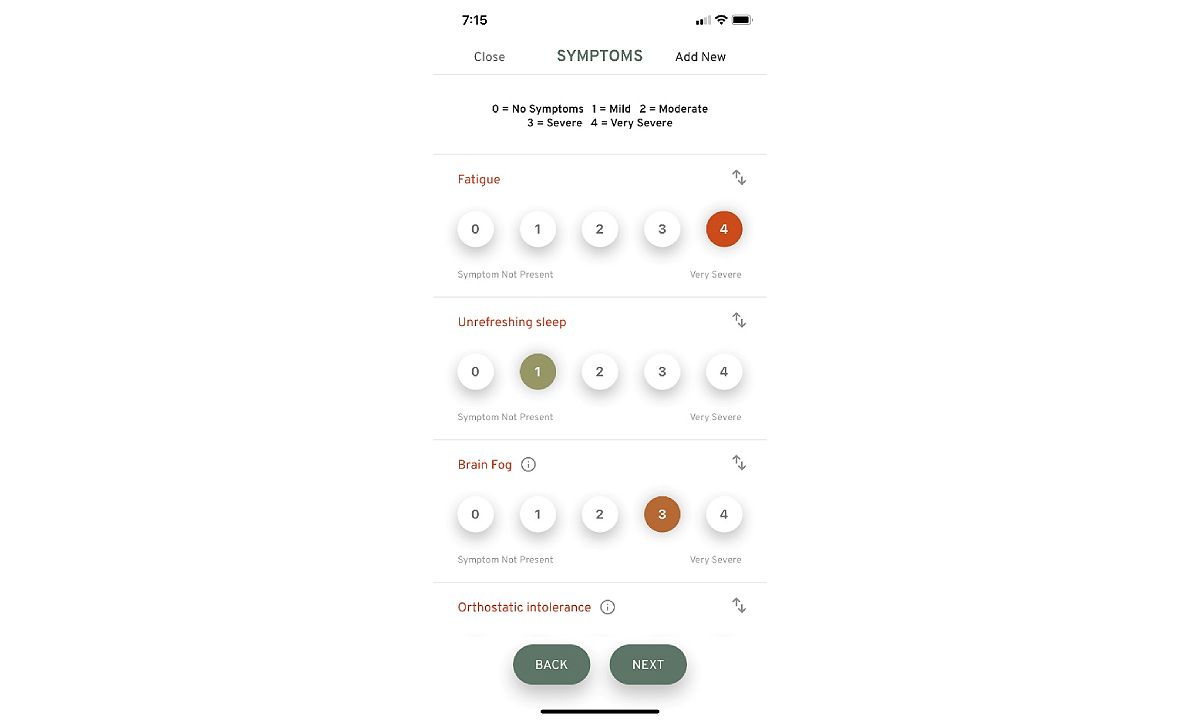
Using the mobile app tracking screen, users can report the presence and severity of symptoms felt.

**Figure 5:**
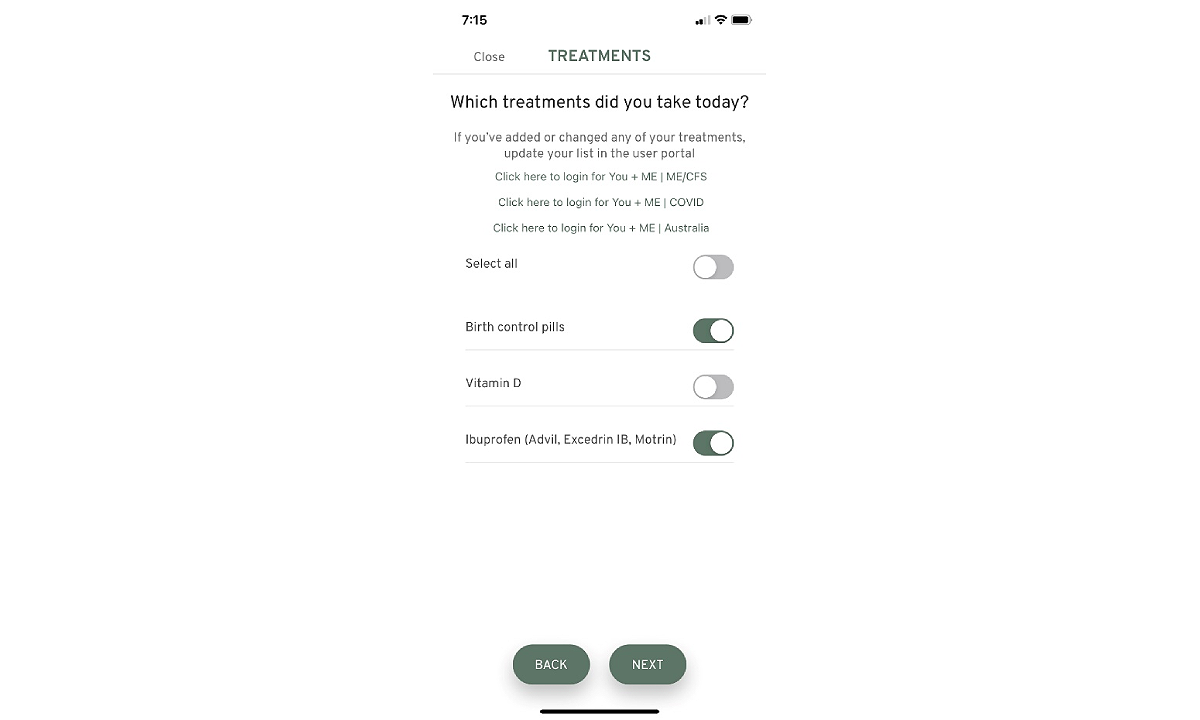
Using the mobile app tracking screen, users can log which treatments were taken that day. ME/CFS: myalgic encephalomyelitis/ chronic fatigue syndrome.

**Figure 6:**
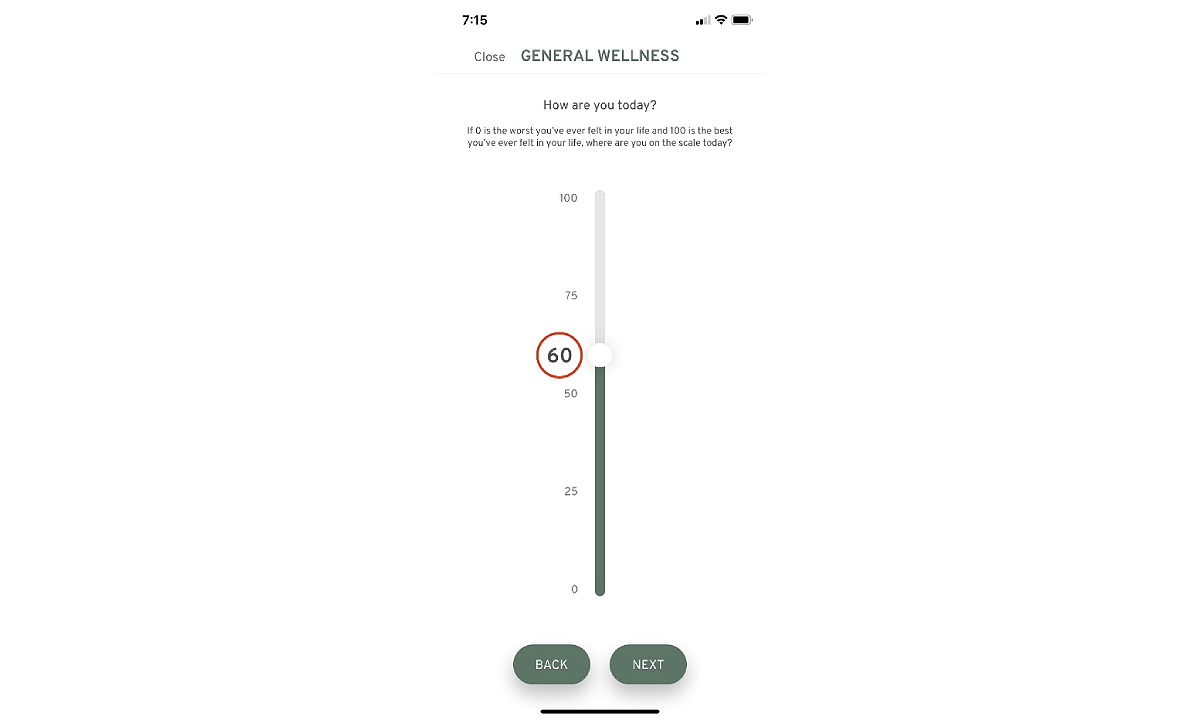
Using the mobile app tracking screen, users can provide a rating of general wellness.

Upon completion of the first set of surveys, participants are sent an email with a link to download the You + ME symptom-tracking mobile app. The symptom-tracking app allows individuals with ME/CFS, LC, and other chronic diseases to record symptoms, lifestyle factors, life events, and any activities on an ongoing basis (Figures 4-6).

### Data Management

The principal investigator is responsible for overseeing data quality and reliability and ensuring safeguards are in place to protect participant privacy and safety. The Registry data manager audits the data biweekly to improve quality, ensure validity and reliability, and guarantee the integrity and credibility of output.

### Data Sharing

Researchers who want to access the data for research apply to Solve M.E. with information required to review their request, including their name, institution, terminal degree, relevant publications, and research interest. The application is reviewed by the You + ME Innovation Council, a group of approximately 12 individuals with deep expertise in ME/CFS and chronic disease research or data science (clinicians, researchers, data scientists, and individuals with ME/CFS and other chronic diseases). Data are stripped of identifiers and shared with researchers through secure means of transfer. Researchers who use the data are required to sign a data use agreement that includes a guarantee to share their methods and findings with the ME/CFS and LC patient communities.

### Ethical Considerations

The Registry was approved and is overseen by the Western Institutional Review Board (Protocol #20193104).

## Results

### Participant Enrollment

As of September 30, 2021, the Registry had over 4200 participants: 3033/4339 (69.9%) people with ME/CFS, 833/4339 (19.2%) post–COVID-19 people, and 473/4339 (10.9%) control volunteers. Recruitment to the Registry continues at a steady pace, with an average of 72 new people registered every week, responding to social media and other outreach efforts.

**Figure 7:**
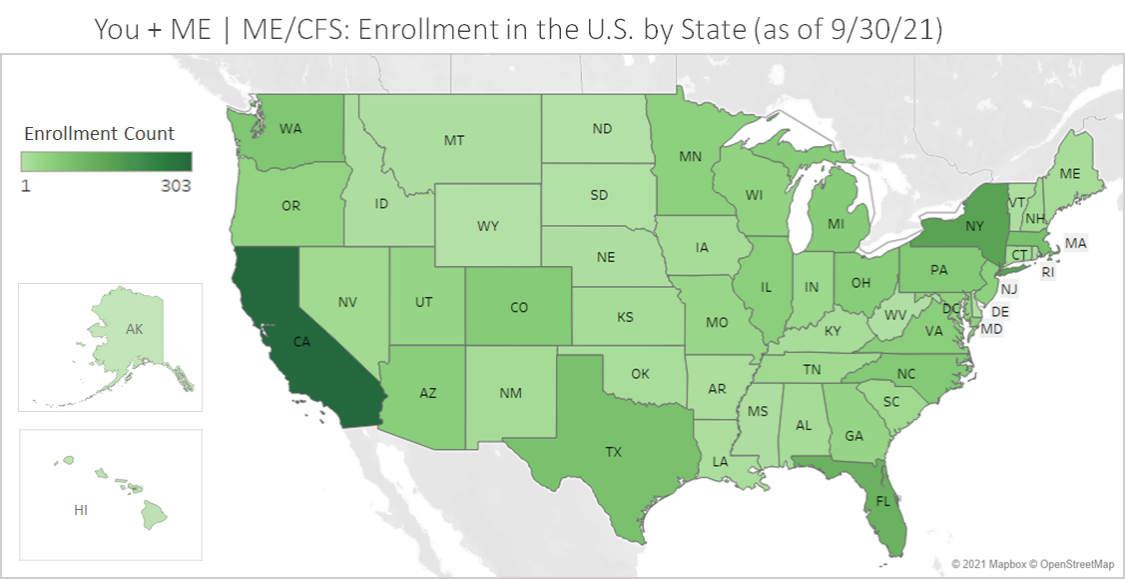
Registry enrollment of adults with ME/CFS in the United States by state (as of September 30, 2021). Map based on longitude (generated) and latitude (generated). Each state with ME/CFS and control volunteers enrolled shows a color corresponding to enrollment count aggregated from zip code data provided by participants (N=2085). ME/CFS: myalgic encephalomyelitis/ chronic fatigue syndrome; Registry: You + ME Registry and Biobank.

**Figure 8:**
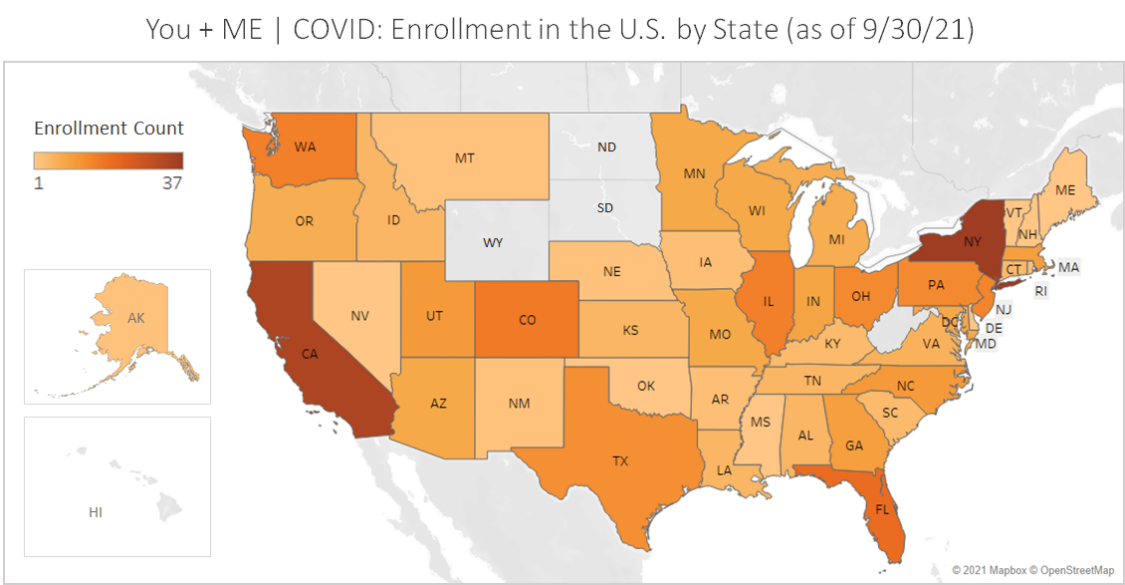
Registry enrollment of adult control volunteers in the United States by state (as of September 30, 2021). Map based on longitude (generated) and latitude (generated). Each state with participants enrolled shows a color corresponding to post–COVID-19 enrollment count aggregated from zip code data provided by the participants (N=387). ME: myalgic encephalomyelitis; Registry: You + ME Registry and Biobank.

**Figure 9:**
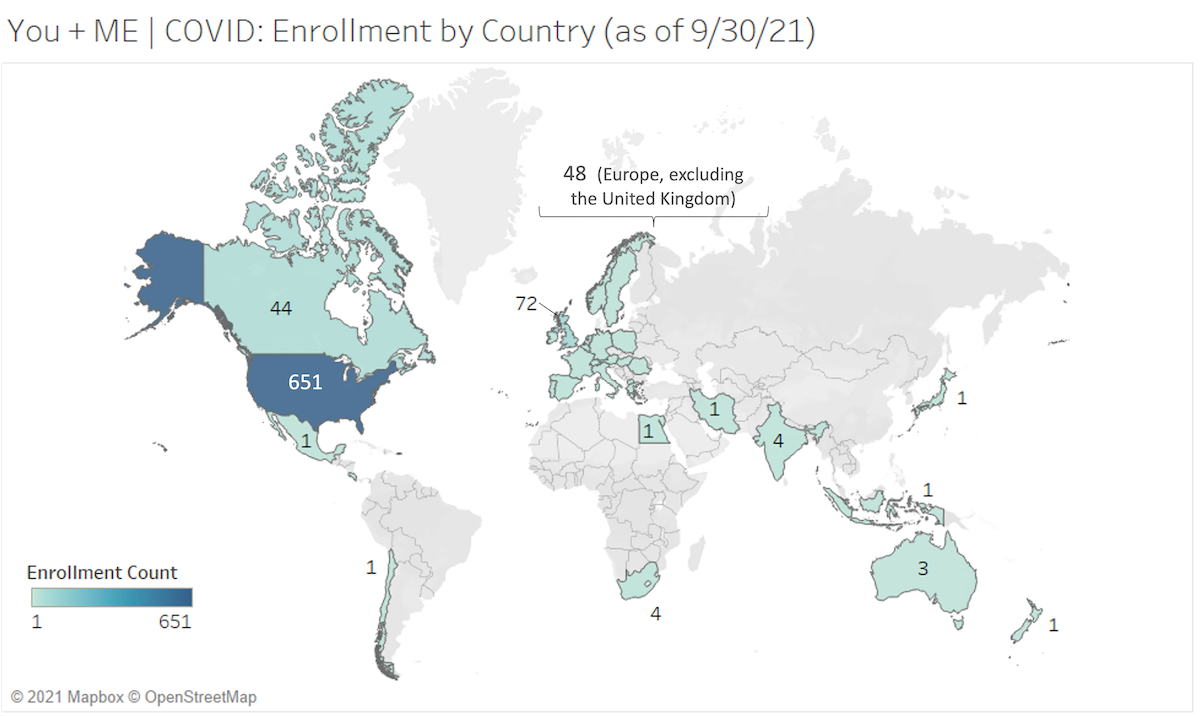
Registry enrollment of adults post–COVID-19 by country (as of September 30, 2021). Map based on longitude (generated) and latitude (generated). Each country with participants enrolled shows a color corresponding to post–COVID-19 enrollment count from country of residence data provided by the participants (N=836). ME: myalgic encephalomyelitis; Registry: You + ME Registry and Biobank.

The Registry includes participants from all 50 states of the United States (see Figures 7 and 8). Participant-provided zip codes matched to rural-urban commuting area codes from the 2010 Census indicate that 256/2085 (12.3%) of ME/CFS and 42/387 (10.9%) of LC registrants who provided zip code data live in nonurban areas. The Registry is open to LC registrants internationally. The highest concentration of LC registrants is in the United States (n=651, 78.2%), the United Kingdom (n=44, 5.3%), and Canada (n=72, 8.6%); in total, there is representation from 32 countries (see Figure 9).

Although a major goal of the Registry is to open up participation in research to underrepresented groups, enrollment to date is largely consistent with previous ME/CFS research cohorts. The male-to-female sex ratio for the entire Registry cohort is 23:100, meaning for roughly every 23 males, there are 100 females (intersex individuals make up 0.001% of the participants). Registry participants are predominantly non-Hispanic White. An area of divergence from most previous studies is the significant proportion of individuals in the Registry who are severely-to-very-severely ill, including nearly one-third (581/1096, 30.5%) of our ME/CFS cohort and 123/782 (15.7%) of our post–COVID-19 cohort who have provided a response to the Karnofsky Performance Status survey. These patients, who are house- or bed-bound, are underrepresented in traditional research settings.

Overall enrollment targets by cohort for the first 3 years of the Registry are summarized in Table 4. The goal is for 30% of the ME/CFS and post–COVID-19 cohorts to be controls (in the post–COVID-19 cohort, controls are individuals who had COVID-19 but fully recovered and never experienced LC). Another goal is for 25% of the entire Registry cohort to be based outside the United States by the end of year 3.

**Table 4:** Registry^a^ enrollment targets by cohort for years 1-3.

Cohort	Year 1 (N=3800), n (%)	Year 2 (N=9000), n (%)	Year 3 (N=15,000), n (%)
ME/CFS^b^ cohort^c^	3000 (78.9)	7000 (77.8)	10,000 (66.7)
LC^d^ cohort^e^	800 (21.1)	2000 (22.2)	5000 (33.3)

^a^Registry: You + ME Registry and Biobank.

^b^ME/CFS: myalgic encephalomyelitis/chronic fatigue syndrome.

^c^Includes control volunteers without ME/CFS.

^d^LC: long COVID.

^e^Includes controls (COVID-19-recovered).

### Mobile App Engagement

According to an analysis completed in August 2021, 1358 participants (56.1% of those who had enrolled by the end of July 2021) with ME/CFS and LC had downloaded the mobile tracking app and over 1264 (93.1% of those who had downloaded the app) initiated tracking. Nearly half of the app users had tracked symptom and other health data on 10 or more days. Collectively, mobile app users had logged 38,242 tracking days.

**Figure 10:**
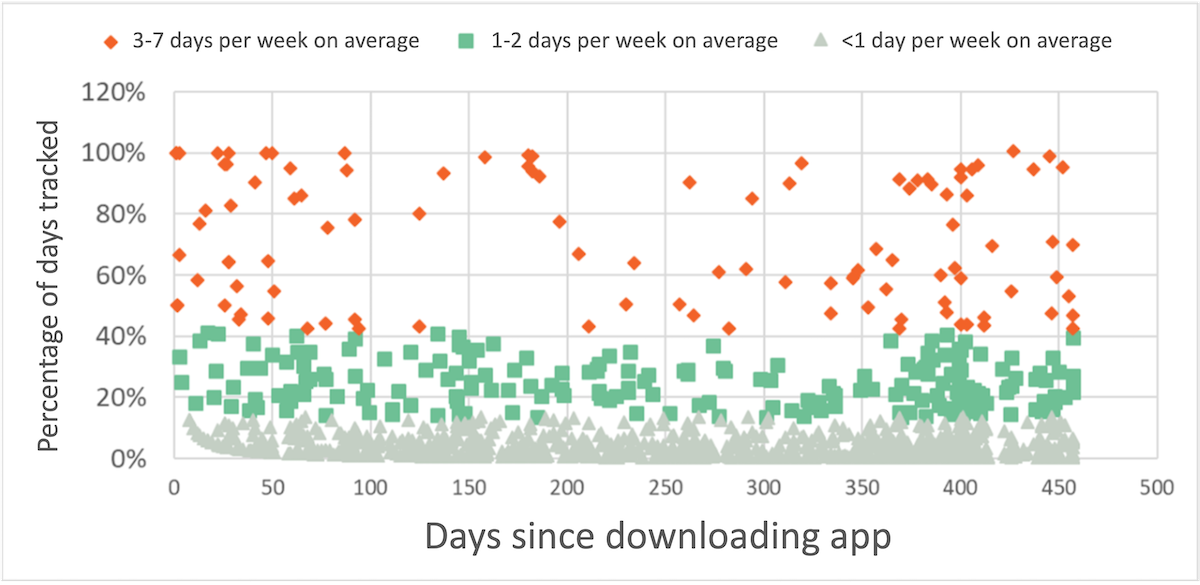
Percentage of days tracked per week (on average) by each symptom-tracking mobile app user since date of app download. The percentage of days tracked were bucketed into three categories and color-coded in the graph: high engagement (3-7 days per week) moderate engagement (1-2 days per week) and low engagement (less than 1 day per week).

The days per week tracked by users (on average) showed high engagement (3-7 days per week) from a superuser group comprising 106 (8%) of users. In addition, 207 (16%) had moderate engagement (1-2 days per week), and three-quarters (n=951) had low engagement (less than 1 day per week); see Figure 10.

### Participant Satisfaction

**Figure 11:**
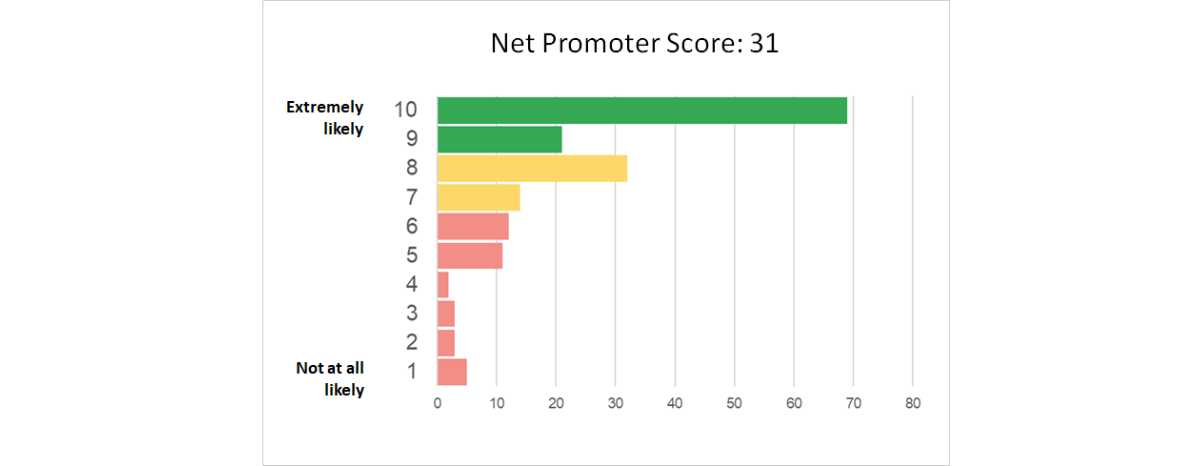
You + ME user satisfaction index measured by the net promoter score (as of September 30, 2021). ME: myalgic encephalomyelitis.

Of 172 participants who completed our community feedback survey as of September 30, 2021, 122 (70.9%) rated their overall satisfaction with the Registry as 7 or higher (on a scale of 1-10). The Registry has qualified as “great” using a user satisfaction index measurement called a net promoter score, in which respondents are asked how likely they are to recommend it to a friend (see Figure 11).

## Discussion

### Preliminary Results: Participant Enrollment and Satisfaction

This paper describes the design, operation, recruitment, geographical coverage, and overall participant satisfaction for the Registry. With a plan to enroll a large and diverse cohort for longitudinal data collection, this data resource has the potential to improve the understanding of ME/CFS and LC, facilitate translational research, and inform clinical care and improve the quality of life for people living with ME/CFS and LC.

Resources such as the UK Biobank and other large-scale research efforts have demonstrated the power of large cohorts for biomedical discovery [48] and motivated our goal to collect data from tens of thousands of individuals. Enrollment in the Registry has been robust, with over 4000 participants registered within 2 years of opening; participant accrual and the trajectory toward enrollment targets have been consistent with similar online disease registries [15,16].

Compared to traditional, in-person studies, online studies and web-based recruitment can enable participation from people with diverse geography, backgrounds, and disease experiences [15-17]. The Registry has successfully enrolled some hard-to-reach participants, including people from nonurban areas and severely ill patients, who are house- or bed-bound. In addition to the convenience of online participation, strong enrollment in the Registry is likely driven by the broad eligibility criteria and highly motivated patient populations who want to contribute to research [49] that will help them and others, as has been observed in other disease registries.

Users have reported finding the mobile app useful for ongoing tracking of their symptoms and other factors, but use of the app is extremely variable. Others only use it for a limited period due to a multitude of factors, including the burden of frequent tracking, mobile app functionality (eg, periodic bugs or personal preferences around app functionality), or an aversion to being reminded of their health. Deploying strategies that encourage more frequent tracking with the app is an ongoing priority.

The high degree of satisfaction with both the data collection process and the digital tools reported by the You + ME community members reflects the incorporation of perspectives from multiple stakeholders into the Registry design and the benefits of cocreation and HCD in the development of digital health tools [28,50-52]. Facilitating a mechanism for those living with an illness to share the unique insights of their lived experience and partnering with them on the development of tools to capture that experience are best practices that should be universally adopted to improve our understanding and therapeutic development.

Participant satisfaction and commitment to the Registry will be key to continued success and further expansion. We learned from other efforts that a meaningful approach to maximizing retention and engagement in a research study over time is sharing individualized data and research results back [53,54]. You + ME registrants have access to the survey information they provide, as well as readouts of symptom data from the tracking app that provide a resource for self-management and sharing of information with health care providers and loved ones.

### Using the Data to Drive Research

The overarching goal of the Registry is to serve as a catalyst for critical research into diagnostics, treatments, and cures for ME/CFS, LC, and other postinfection diseases using the power of a large cohort with prospectively collected data. The data collection combines patient-reported outcomes that are important to community members with validated scales. These data can be used for researching associations between numerous characteristics and disease courses and to validate outcome data reported by patients as benchmarks for future approval of treatments [15,16].

One of the benefits of this data set is the ability to look across cohorts to understand the similarities and differences between ME/CFS and LC. Cross-disease and disease subtype comparisons using data from registries have produced valuable insights, including clarification of clinical profiles and implication of targeted therapies [55,56]. The establishment of the Registry during the COVID-19 pandemic, during which case numbers have been highly concentrated over a short period, presented an opportunity to track and study trajectories of symptom improvement or worsening in a population with a singular infectious trigger. Clues about susceptibility and resilience to long-term effects of COVID-19 could also benefit the millions of Americans already diagnosed with ME/CFS [56].

The Registry has started to support data analysis by the internal research team and in partnership with external researchers. One example of active promotion of the Registry is through the Solve M.E. Ramsay Grant Program, an annual peer-reviewed competition for grants in support of pilot studies that first launched in 2016. In 2021, the Ramsay Grant Program opened a new funding mechanism to analyze Registry data, ultimately funding 2 projects [5].

To further increase accessibility and utility of the Registry research data set, we plan to build an interface that allows vetted researchers to query and use the data for a range of scientific projects. Researchers will be required to share their results so a community of researchers can further test the findings or build from them in new work.

### Limitations

Despite these successes, the current Registry study design and data set has some limitations, including reliance on self-report data, which can produce a measurement error due to participant recall, interpretation, or other factors [57]. The Registry is also subject to selection bias, including sociodemographic and other differences between participants and nonparticipants, selective participant drop-off, and missing data. These are frequent concerns in registry-based research and introduce data accuracy, interpretation, and generalizability issues. We are therefore planning a number of actions to improve the representativeness of the Registry cohort and pursuing studies to evaluate our measurements, guide development of the protocol, and examine the quality and usefulness of the data.

#### Engagement and Data Completeness

Both ME/CFS and LC are illnesses that evolve and change over time. Although the Registry and symptom-tracking app are specifically designed to capture this, they are dependent on continued engagement from the Registry community, both within visits and over time. Data completeness, potential bias in completion rates, and data quality will be continuously monitored. Developing novel approaches for engagement and learning from others [50,52] who have successfully achieved this will be an ongoing priority.

#### Diversifying the You + ME Community

The Registry is predominantly made up of White non-Hispanic individuals. Ethnic minorities are underrepresented as participants in biomedical and public health research, due to a multitude of personal (eg, cultural distrust and perceptions of research), social (eg, expenses, work, and home responsibilities), and research-related (eg, inaccessibility of study documents and materials, travel to study locations) factors [58]. The use of online surveys that can be completed at home addresses some of these barriers to participation, but there are still reported ethnic and socioeconomic status (SES) differences in web-based research study participation [59]. In collaboration with partners, a directed effort will be made to increase Registry inclusivity and participation and to develop strategies that address recruitment bias.

#### Expanding Our Control Cohort

The existing control cohort represents a little more than 10% of overall participants; the target is 30%. To ensure the control cohort is adequately matched on key demographic variables and therefore able to serve as a comparison group to our ME/CFS and LC cohorts, direct, targeted outreach and more innovative approaches, including partnership with other disease registries to share a control data set, will be explored.

### Next Steps for the Registry

#### Meeting the Needs of Adolescents

Both ME/CFS and LC affect adolescents; this group is often underrepresented in clinical research [60-62]. The symptom clusters experienced by this population are often distinct from the adult population; for example, many adolescents with ME/CFS have OI as a predominant symptom [63,64].

Development of a version of the Registry for adolescents aged 13-17 years is currently underway. It will be designed specifically for this age group, so it includes an appropriate consent and data collection process.

#### Biosample Collection

To accompany the rich longitudinal phenotypic data collected in the Registry, biological samples will be collected from a subset of the larger Registry cohort to both support specific research projects and create a biorepository of samples for future research. Samples will include 1 or more of the following:

- Dried blood spot (DBS) cards (DNA, RNA, protein expression, and metabolomics analyses)
- Dried urine strips (DUS; metabolomics)
- Fecal samples for analyses of microbiome composition and metagenomics (determination of potential microbial metabolites that affect gastrointestinal, immune, metabolic, neurologic, and systemic health)
- Saliva (salivary biomarkers)
- Venipuncture blood draw, and processing and storage of blood components (immunologic, metabolomics, microbiome/virome)

These sample types can support a range of research and make up the immediate biosample collection protocol, but studying other tissue types, such as cerebrospinal fluid, is possible in the future. Samples will be stored by a certified good clinical practice (GCP) provider indefinitely in the Solve M.E. Biobank but destroyed upon request if a participant withdraws.

#### Expanding the Registry to Integrate New Data Types

Although the current capabilities of the Registry can support expansive data collection, the platform is also built with the capacity to integrate new data types (eg, passive monitoring from wearables and health care data from participants’ clinicians). This gives great potential for multimodal data, in particular physiological data, that can be combined with self-reported data to significantly increase the accuracy and validity of results and to reduce bias.

## Abbreviations

HCD: human-centered design
LC: long COVID
ME/CFS: myalgic encephalomyelitis/ chronic fatigue syndrome
OI: orthostatic intolerance
PEM: postexertional malaise
Registry: You + ME Registry and Biobank
Solve M.E.: Solve ME/CFS Initiative
UKMEB: UK ME/CFS Biobank
