# Correction: The Facilitation of Clinical and Therapeutic Discoveries in Myalgic Encephalomyelitis/Chronic Fatigue Syndrome and Related Diseases: Protocol for the You + ME Registry Research Platform

**DOI:** 10.2196/42030

**Published:** 2022-10-13

**Authors:** Allison Ramiller, Kathleen Mudie, Elle Seibert, Sadie Whittaker

**Affiliations:** 1 Solve ME/CFS Initiative Glendale, CA United States

In “The Facilitation of Clinical and Therapeutic Discoveries in Myalgic Encephalomyelitis/Chronic Fatigue Syndrome and Related Diseases: Protocol for the You + ME Registry Research Platform” (JMIR Res Protoc 2022;11(8):e36798) the authors noted an error in the final paragraph of the Acknowledgments section.

In the originally published article, the final paragraph of the Acknowledgments section was published with an incorrect grant number as follows:

“The development of the Registry database and mobile app was supported in part by the National Institutes of Health (NIH) grant U24-NS-105525 as part of the ME/CFS Collaborative Research Network.”

The final paragraph of the Acknowledgments section has been updated with the correct grant number (U24-NS-105535), to read:

“The development of the Registry database and mobile app was supported in part by the National Institutes of Health (NIH) grant U24-NS-105535 as part of the ME/CFS Collaborative Research Network.”

The correction will appear in the online version of the paper on the JMIR Publications website on October 13th, 2022, together with the publication of this correction notice. Because this was made after submission to PubMed, PubMed Central, and other full-text repositories, the corrected article has also been resubmitted to those repositories.

